# Diversity, Equity, and Inclusion in the Microbial Sciences—the Texas Perspective

**DOI:** 10.1128/mBio.02620-21

**Published:** 2021-10-19

**Authors:** Alfredo G. Torres, Maria Elena Bottazzi, Floyd L. Wormley

**Affiliations:** a University of Texas Medical Branch, Department of Microbiology and Immunology, Galveston, Texas, USA; b Baylor College of Medicine, Department of Pediatrics, Molecular Virology & Microbiology, National School of Tropical Medicine, Houston, Texas, USA; c Texas Christian University, Department of Biology, Fort Worth, Texas, USA

**Keywords:** diversity, equity, inclusion

## Abstract

The way that diversity, equity, and inclusion impact scientific careers varies for everyone, but it is evident that institutions providing an environment where being different or having differences creates a sense of being welcomed, supported, and valued are beneficial to the scientific community at large. In this commentary, three short stories from Texas-based microbiologists are used to depict (i) the importance of bringing the guiding principles of diversity, equity, and inclusion within their professional roles, (ii) the need to apply and translate those principles to support and enable successful scientific careers among peers and trainees, and (iii) the impact of effective science communication to increase the understanding of microbial environments among the community at large.

## COMMENTARY

Many higher education institutions and scientific societies nationwide, including the American Society for Microbiology, have made a pledge to foster diverse, equitable, and inclusive environments that value and embrace the different ethnicities, cultures, religions, and identities that represent their members. Nationwide, institutions and societies are implementing those principles based on the community they serve, and Texas is not the exception. It has been estimated that by 2020, blacks and Hispanics comprised over half of Texas’s total workforce; unfortunately, both groups are significantly underrepresented in the state’s higher education institutions, particularly in STEM disciplines (<10% of the workforce). More alarming is the lack of professors of color who can serve as role models for those individuals who want to join scientific disciplines. Here are three short stories of Texan microbiologists trying to make a difference in their community and embracing diversity, equity, and inclusion in their professional careers.

### Hispanic leadership in vaccine sciences while advocating for women in biotechnology (Dr. Maria Elena Bottazzi).

“Everything is Bigger in Texas,” including the home of the world’s largest medical center, located in Houston. The Texas Medical Center, with more than 50 member institutions, all working together delivering health care and promoting innovation in research and education, is my professional home. I am a Hispanic professor, vaccine scientist, microbiologist, and global health advocate affiliated with Baylor College of Medicine and Texas Children’s Hospital and with multiple secondary appointments at other Texas-based institutions.

My scientific interests, having grown up and trained as a microbiologist in Honduras, revolve around tropical medicine or the study of neglected tropical diseases and related conditions which affect disproportionally the health and social and economic status of the world’s poorest, approximately 10% of the global population. Today, I lead the Section of Tropical Medicine in the Department of Pediatrics and co-lead the Texas Children’s Hospital Center for Vaccine Development, with the mission of increasing Hispanic presence in the vaccine sciences and applying diversity, equity, and inclusion (DEI) guiding principles. The Vaccine Center is composed of a diverse and multidisciplinary team of faculty, scientists, and staff with a portfolio of programs that develop safe, effective, and affordable vaccines as biotechnology solutions to prevent or treat these diseases. Not only do our teams and global partners have different ethnicities, cultures, religions, and identities, but importantly, our research programs are designed to conform to the needs, customs, and traditions of the people whom the vaccine products are intended to benefit. The goal is to decolonize the vaccine development process by building and strengthening local capacity for self-sufficiency.

It has not been easy, as a Hispanic woman, to build a career and climb the health sciences academic ranks, while establishing nontraditional biotechnology collaborating hubs by leveraging the academic structures. It required me to be bold, flexible, and resilient. It required me to think “out of the box” and go beyond areas of my own comfort zone. I had to learn how to shift from a transactional leadership mentality into using transformational inclusive and adaptive leadership principles. Therefore, my personal goal is to continue implementing DEI principles and advocate for, engage, motivate, and empower young, especially women, scientists so that they themselves can diversify biotechnology innovations, catalyze policies, and disseminate trustworthy science information.

### Views from one African American biomedical scientist (Dr. Floyd L. Wormley, Jr.).

A robust community of biomedical scientists and clinicians requires representation from all demographics of our population. This means recruiting a diverse population of students into our graduate programs and taking the necessary steps to ensure inclusive and equitable training opportunities. The reliance on standardized tests, exams that do not measure an individual’s “grit,” for admission decisions appears to be on the decline. However, holistic application reviews are still susceptible to reviewer bias against applicants from non-research-intensive universities and/or institutions with predominantly underserved and underrepresented student populations as some reviewers may view applicants from these institutions as not equipped for the rigors of graduate or professional school. Graduate students and faculty who are women and/or members of underrepresented groups in the biomedical sciences should not be the only ones “taxed” for outreach efforts to other underserved populations; outreach is the responsibility of us all.

I continue to benefit from the wisdom of incredible mentors who modeled success, supported my professional and personal growth, and guided me throughout my journey. However, we suffer from a shortage of underrepresented minority mentors in the professorate and biomedical sciences. The impact of this shortage is clearly seen as we are experiencing a time when their voices are desperately needed for outreach to populations that, due to historical failures of the public health system, do not trust our public health officials. Also, not all career tracks will, or should, end with a doctorate and a tenure-track academic position. Our training programs should prepare biomedical scientists for a multitude of careers and broaden their indicators of success so as not to discourage the participation of trainees interested in careers outside academia.

I remain hopeful that our combined efforts will result in a diverse, inclusive, and equitable biomedical workforce that will serve everyone no matter their socioeconomic status, ethnicity, or sexual identification. These categories do not matter to pathogens and should not matter to us.

### Understanding pathogenic Escherichia coli as a common goal of the Hispanic/Latino community (Dr. Alfredo G. Torres).

As a Hispanic faculty member at the University of Texas Medical Branch, I have been interested in diseases that affect developing countries and used my research program to promote the advancement of junior scientists, particularly those from underrepresented backgrounds. One area of interest in my laboratory has been trying to understand the pathogenic processes that some isolates of Escherichia coli use to cause diarrheal disease. Interestingly, diarrhea caused by pathogenic E. coli remains a significant cause of morbidity and mortality in developing countries, including in Latin America.

As I advanced in my academic career, I was able to establish multiple collaborations with investigators from Latin America who share the same interest in pathogenic E. coli, and together we were able to train several talented junior investigators. However, during that time, it was clear that a lot of the research efforts in that region were impacted by the lack of funding and resources and, eventually, the difficulty in publishing. Investigators in Latin America are as diverse as the strains of pathogenic E. coli. So, the challenge was to create an organization in which all these scientists from different backgrounds were able to work together for common goals, advancing the understanding of pathogenic E. coli in Latin America, promoting collaboration and sharing research information within laboratories of the region, increasing the education of the next generation of researchers, and enhancing the number of collaborative publications.

As a result, in 2009, the Latin American Coalition for Escherichia coli Research (LACER) was launched and currently encompasses 70 research groups in 11 Latin American countries and the United States. LACER practices DEI principles, from including a diverse group of investigators in different disciplines complementary to study E. coli, to the inclusion of young and senior investigators in the planning and execution of research activities, training, and writing of grants, manuscripts, and books. As we have been able to advance DEI in LACER, it is evident that such principles can be incorporated in any institution or association.

### Closing remarks.

These short essays are intended to demonstrate the experiences of three Texas-based scientists in their academic paths and provide some topics for conversation regarding DEI principles. The urgent national challenge to diversify the scientific workforce is real, and research universities, academic medical centers, and scientific societies need to participate in this process while ensuring scientific excellence and advancing equity across higher education and the biomedical professions.

